# Supplementary material for: Enhanced Power Factor and Ultralow Lattice Thermal Conductivity Induced High Thermoelectric Performance of BiCuTeO/BiCuSeO Superlattice
Source: Materials (Basel). 2023 Jun 11;16(12):4318. doi: 10.3390/ma16124318 (PMC10305665; doi:10.3390/ma16124318)
Supplement: Supplementary file 1 [file materials-16-04318-s001.zip › materials-2407671-supplementary.pdf]

Enhanced power factor and ultralow lattice thermal conductivity induced high thermoelectric performance of BiCuTeO/BiCuSeO superlattice

Xuewen Yang[a,b], Zhiqian Sun[a,b], Guixian Ge*[a,b], Jueming Yang*[a,b]

[a]College of Science/Xinjiang Production & Construction Corps Key Laboratory of Advanced Energy Storage Materials and Technology, Shihezi University, Shihezi City 832000, China

[b]Xinjiang Production and Construction Corps Key Laboratory of Oasis Town and Mountain-basin System Ecology, Shihezi University, Shihezi City 832000, China

*Corresponding authors: geguixian@126.com (G. Ge); juemingyang11@gmail.com (J. Yang);

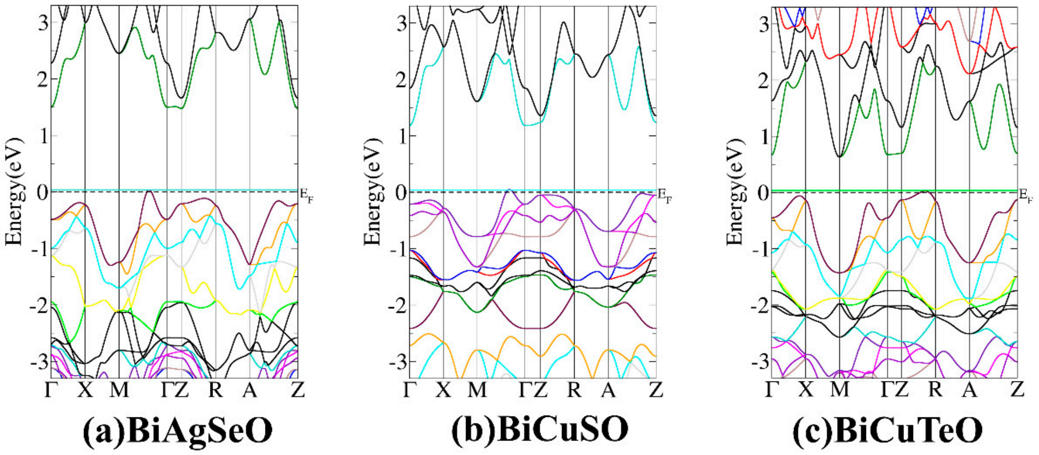

Fig. S1 The band structures of BiMChO.

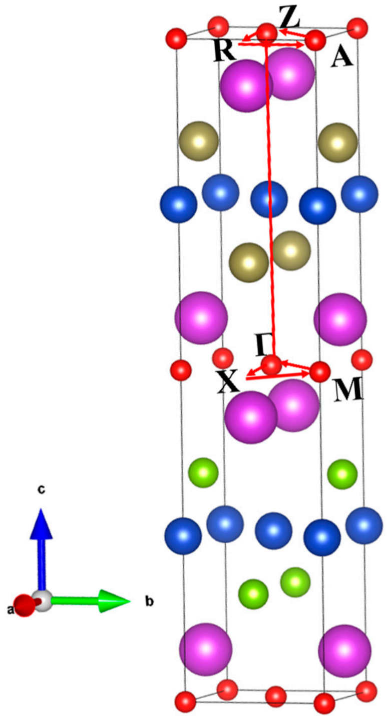

Fig. S2 The high symmetry points of BiMChO superlattices.
